# Azacitidine-Venetoclax in Elderly and Unfit Acute Myeloid Leukemia: Real-World Outcomes Outside Clinical Trials

**DOI:** 10.3390/hematolrep18050073

**Published:** 2026-09-30

**Authors:** Manlio Fazio, Demetrio Gabriele Gerace, Giuseppe Mirabile, Rossana Leanza, Giuseppa Penna, Patricia Rizzotti, Sabina Russo, Adele Bottaro, Maria Elisa Nasso, Noemi Di Martino, Fabio Stagno, Alessandro Allegra

**Affiliations:** 1 Division of Hematology, AOU Policlinico “G. Martino”, Via Consolare Valeria 1, 98125 Messina, Italy; 2 Department of Human Pathology in Adulthood and Childhood “Gaetano Barresi”, University of Messina, Via Consolare Valeria 1, 98125 Messina, Italynoemi.dimartino.2001@gmail.com (N.D.M.)

**Keywords:** acute myeloid leukemia, venetoclax, hypomethylating agents, molecular risk classification, fitness, augmented HCT-CI, myelodysplasia-related AML

## Abstract

**Background:** Azacitidine plus venetoclax (AZA+VEN) is the current standard first-line treatment for elderly or medically unfit patients with acute myeloid leukemia (AML). However, patients treated in real-world settings often differ from those enrolled in clinical trials due to frailty, comorbidities, secondary AML, and prior exposure to hypomethylating agents. **Materials and Methods**: We retrospectively analyzed 21 consecutive AML patients who received frontline AZA+VEN between October 2020 and May 2026. Patient fitness was assessed according to SIE/SIES/GITMO criteria. Data on treatment responses, toxicities, treatment modifications, survival outcomes, ELN 2022/2024 risk categories, and comorbidity burden, including HCT-CI and augmented HCT-CI scores, were collected. **Results**: Median age was 77.2 years, and 95.2% of patients were considered unfit for intensive chemotherapy. Secondary AML was observed in 42.9% of cases, including 23.8% arising from myelodysplastic syndromes (MDSs) previously treated with azacitidine. The overall response rate was 57.1% (12/21), with a composite complete remission (CR/CRi) rate of 42.9%. The median time to response was 1.8 months. Venetoclax schedule modifications were required in 66.7% of patients, while azacitidine dose/schedule reductions were necessary in 57.1%. The augmented HCT-CI was significantly associated with OS, supporting further investigation of its potential prognostic value in this setting. **Conclusions**: AZA+VEN demonstrated meaningful clinical activity and manageable toxicity in elderly or unfit AML patients. Frailty and comorbidity burden had a substantial impact on outcomes, supporting the integration of biological and fitness-based risk assessment in the management of AML in real-world clinical practice.

## 1. Introduction

Acute myeloid leukemia (AML) predominantly affects elderly patients, with a median age at diagnosis of 68 years, many of whom are not candidates for intensive induction chemotherapy (CHT) because of advanced age, frailty, comorbidities, or poor performance status (PS) [1]. Historically, therapeutic options for these patients were limited and associated with poor survival outcomes [2]. The introduction of venetoclax (VEN), a selective BCL-2 inhibitor, in combination with hypomethylating agents (HMAs), has significantly changed the therapeutic landscape for newly diagnosed AML patients considered unfit for standard induction therapy [2]. The phase III VIALE-A trial demonstrated superior overall survival (OS) and overall response rates (ORR) with venetoclax plus azacitidine (AZA) compared with AZA alone, establishing AZA+VEN as the current standard of care for unfit AML patients [3]. Additional studies, including VIALE-C and subsequent real-world experiences, have further supported the efficacy of venetoclax-based regimens in this population [4]. Nevertheless, patients meeting eligibility criteria for enrollment in registrational trials often differ substantially from those treated in routine clinical practice, particularly with respect to advanced age, comorbidity burden [5], and prior exposure to hypomethylating agents. Secondary AML arising from myelodysplastic syndromes (MDSs) or myeloproliferative neoplasms (MPNs) represents a biologically adverse subgroup frequently encountered in real-world settings [6,7,8]. The optimal management of patients previously exposed to AZA for antecedent MDS remains challenging, and data regarding the efficacy of subsequent AZA+VEN therapy are still limited. Furthermore, the recent introduction of the ELN 2024 classification has refined prognostic stratification beyond the established ELN 2022 system, potentially improving risk assessment and the interpretation of treatment outcomes in venetoclax-treated patients [9]. In this context, accurate assessment of biological fitness is crucial, as chronological age alone inadequately reflects treatment tolerance and prognosis. Several tools have been developed to quantify the impact of comorbidities, among which the Hematopoietic Cell Transplantation–Comorbidity Index (HCT-CI) is one of the most widely validated instruments [10]. More recently, the augmented HCT-CI, which incorporates serum albumin, ferritin, and platelet count, has demonstrated improved prognostic discrimination compared with the standard HCT-CI [11]. Growing evidence suggests that comorbidity burden and biological fitness may influence outcomes independently of disease-related risk factors, highlighting the importance of comprehensive patient assessment in contemporary AML management. On this basis, we conducted a single-center retrospective real-world analysis of AML patients treated frontline with AZA+VEN who were considered ineligible for intensive chemotherapy according to the SIE/SIES/GITMO criteria. The study aimed to characterize treatment efficacy, safety, and survival outcomes in routine clinical practice and to explore the prognostic impact of ELN 2022 and ELN 2024 risk stratification, molecular features, prior exposure to azacitidine for antecedent MDS, and patient fitness as assessed by the HCT-CI and the augmented HCT-CI.

## 2. Patients and Methods

**Figure 1:**
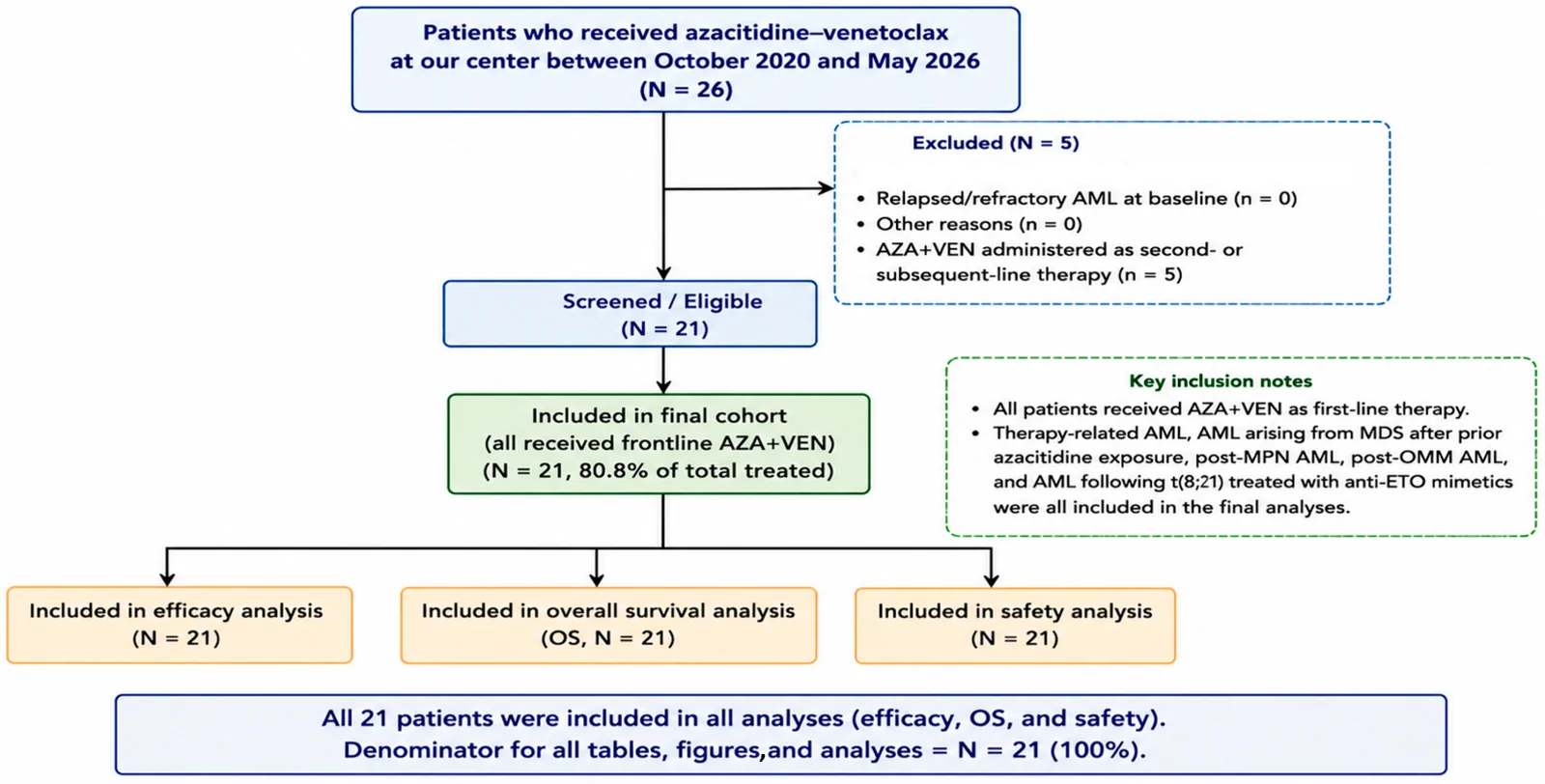
Cohort flow diagram. The diagram shows the number of patients screened and included in the retrospective study, the eligibility criteria, exclusions, and the number included in each analysis.

We conducted a retrospective, single-center, real-world study including consecutive patients diagnosed with acute myeloid leukemia (AML) and treated with first-line azacitidine plus venetoclax (AZA+VEN) at the Hematology Unit of Policlinico “G. Martino”, Messina, Italy, between October 2020 and May 2026. During the study period, 26 patients received AZA+VEN at our institution. Five patients were excluded because AZA+VEN was administered as second- or subsequent-line therapy for AML. The final study cohort therefore comprised 21/26 (80.8%) consecutive patients treated with AZA+VEN in the frontline setting. No patients meeting this exclusion criterion were identified during the study period; therefore, all 21 screened patients were eligible and included in the final cohort. No patient had relapsed or refractory AML at treatment initiation. Unless otherwise specified, descriptive, efficacy, safety, and survival analyses were performed in the entire study population (*N* = 21) (Figure 1). For subgroup analyses, the corresponding subgroup denominator is reported explicitly in the text, tables, and figures. Eligibility for non-intensive treatment was established according to the SIE/SIES/GITMO (Ferrara) fitness criteria. One patient deemed fit for intensive induction chemotherapy declined standard treatment and was therefore managed with AZA+VEN. AML was classified according to the 2022 WHO classification. Patients without a documented history of an antecedent myeloid neoplasm or prior cytotoxic therapy were categorized as having de novo AML. AML arising in patients with a previously diagnosed myeloid disorder, including myelodysplastic syndromes (MDSs), myeloproliferative neoplasms (MPNs), or MDS/MPN overlap neoplasms, was categorized as secondary AML. Patients with antecedent MDS who had previously received azacitidine remained within the secondary AML subgroup, as prior azacitidine exposure represents treatment of the antecedent disorder rather than a separate disease-origin category. Therapy-related AML was considered separately and defined as AML occurring after prior exposure to cytotoxic chemotherapy and/or radiotherapy for an unrelated malignancy. One patient with de novo AML had a previous history of immune thrombocytopenia (ITP), reported separately in the table for descriptive purposes only; the patient had received thrombopoietin (TPO)-receptor agonists but no prior cytotoxic therapy and we do not suggest any association between this treatment and AML insurgence. Given the small number of patients within each disease-origin subgroup, these categories primarily describe the clinical heterogeneity of the cohort rather than support formal prognostic comparisons; subgroup findings should therefore be considered exploratory and interpreted cautiously. Data were retrospectively collected from institutional medical records and all patients gave written informed consent. Baseline variables included age, sex, AML subtype, Eastern Cooperative Oncology Group (ECOG) performance status (PS), comorbidities, complete blood counts (CBC), biochemical parameters, cytogenetic findings, and molecular characteristics. Molecular profiling was performed at AML diagnosis, before initiation of AZA+VEN, using a targeted next-generation sequencing (NGS) myeloid panel (Oncomine™ Myeloid Assay, Thermo Fisher Scientific, Waltham, MA, USA) on the Ion Torrent S5 platform. All 40 genes included in the panel were successfully evaluated in all patients. The assay interrogated the full coding sequence of selected genes and predefined mutational hotspots of the remaining genes. Sequence analysis was performed using Ion Reporter Software (version 5.0). Laboratory quality-control metrics included a mean sequencing depth > 1000×, with 97% of targeted regions covered at ≥100×. The analytical sensitivity of the assay allowed detection of single-nucleotide variants (SNVs) with a variant allele frequency (VAF) > 2%, insertion/deletion variants (indels) with a VAF > 5%, and indels within homopolymeric regions with a VAF > 10%. Regions with suboptimal coverage were interpreted cautiously because of the potential for false-negative results, particularly for low-VAF variants. Variant annotation and interpretation were based on ClinVar and COSMIC according to established recommendations for the clinical interpretation of somatic variants in oncology. Known polymorphisms and variants classified as benign were excluded from reporting. No paired germline sample was analyzed; therefore, a potential germline origin could not be definitively excluded on the basis of NGS testing alone. In addition, *NPM1*, *FLT3*, and *IDH1/2* mutations were assessed by real-time polymerase chain reaction (RT-PCR).

Patients were classified according to ELN 2017 [12], ELN 2022 [13], and ELN 2024 [14] risk categories. In addition, an exploratory prognostic reassessment was performed using the Beat AML 2024 classification model [15]. Beat AML categories were derived from ELN 2022 risk groups and the molecular score proposed by the Beat AML investigators, incorporating *TP53*, *KRAS*, *KMT2D*, and *IDH2* mutational status. Specifically, patients classified as ELN favorable or intermediate risk were assigned to the Beat AML favorable-risk category, whereas ELN adverse-risk patients were further stratified according to a mutation score assigning + 1 point for *TP53, KRAS*, and *KMT2D* mutations and −1 point for *IDH2* mutations. Patients with a cumulative score ≤ 0 were classified as Beat AML intermediate risk, whereas those with a score ≥ 1 were classified as Beat AML adverse risk. ELN 2017 was retained as a historical reference framework, whereas ELN 2022 represented the updated standard risk classification incorporating advances in molecular and therapeutic knowledge. ELN 2024 was applied to explore the recently proposed risk stratification for patients receiving less-intensive therapies. Beat AML 2024 was additionally assessed as an exploratory molecularly informed model. These analyses were intended to examine how evolving, treatment-contextualized risk models redistribute patients and relate to outcomes in a real-world AZA+VEN-treated cohort, rather than to formally compare or validate their prognostic performance. To provide a comprehensive assessment of patient fitness and comorbidity burden, additional geriatric and comorbidity measures were collected, including the Charlson Comorbidity Index (CCI) [16], the Cumulative Illness Rating Scale (CIRS) [17], and Activities of Daily Living (ADL; Barthel Index) [18]. All mentioned scores (CCI, CIRS, ADL, HCT-CI, and augmented HCT-CI) were retrospectively calculated for study purposes from baseline medical records and were not used to determine treatment allocation. Scores were calculated according to their original published definitions using the available clinical, laboratory, and functional data. Baseline assessment was comprehensive because patients received the first AZA+VEN cycle during hospitalization and routinely underwent evaluation of performance and functional status, comorbidities, hematologic and biochemical parameters, and relevant organ function, including cardiovascular and respiratory assessment. Complete data required for calculation of the investigated scores were available for all 21 patients; therefore, no imputation of missing values was performed.

Correlation analyses were performed between HCT-CI, augmented HCT-CI, and these instruments to evaluate the degree of concordance among different approaches to fitness assessment. Given the increasing interest in comorbidity-based prognostic models in older patients with AML, both HCT-CI and augmented HCT-CI were further explored for their association with treatment outcomes. For survival analyses, HCT-CI and augmented HCT-CI were dichotomized according to their respective cohort median values (6 and 8, respectively). Their association with OS was assessed using Kaplan–Meier analyses with graphical comparisons used to examine survival patterns across the respective score categories. Azacitidine was administered at a dose of 75 mg/m^2^ subcutaneously (s.c.) for 7 consecutive days every 28 days. Venetoclax was administered according to the standard AML ramp-up schedule (100 mg on day +1, 200 mg on day +2, and 400 mg on day +3). Venetoclax exposure and azacitidine schedules were individualized according to response status, hematologic recovery, treatment-related cytopenias, infectious complications, concomitant medications, comorbidity burden, and treatment tolerability. Detailed information on treatment modifications, their timing, and the underlying clinical reasons is provided in the Section 4. Dose reductions, treatment interruptions, shortening of venetoclax exposure, growth factor support, and azacitidine schedule modifications were recorded. Treatment response was evaluated according to European LeukemiaNet (ELN) recommendations. Responses were categorized as complete remission (CR), complete remission with incomplete hematologic recovery (CRi), morphologic leukemia-free state (MLFS), partial remission (PR), stable disease (SD), or non-response (NR). MRD status was recorded as a qualifier of CR/CRi rather than as a separate response category. The overall response rate (ORR) was defined as the proportion of patients achieving CR, CRi, MLFS, or PR [13]. Causes of death were classified as AML-related or non-AML-related based on the predominant clinical cause documented in the medical record. Treatment-related mortality was defined separately as death considered directly attributable to treatment toxicity rather than to AML progression or an intercurrent clinical condition. Molecular response assessment was performed on bone marrow samples using mutation-specific molecular assays for abnormalities identified at diagnosis. NPM1 was assessed according to the protocol described by Gorello et al. [19], FLT3-ITD according to Noguera et al. [20], and IDH1/2 by RT-PCR using the EASY PGX system (Diatech). In the present study, the term molecular MRD was used descriptively to indicate molecular clearance, defined as the absence of a previously detectable mutation at follow-up assessment. Although NPM1 represents an established molecular MRD marker [21], emerging evidence also supports the potential utility of IDH1/2 mutations, particularly IDH2, as molecular markers of residual disease [22]. In line with this emerging vision, clearance of IDH1/2 mutations was interpreted as MRD negativity.

Time to response, duration of response, relapse occurrence, and time to relapse were recorded. Adverse events were retrospectively identified through systematic review of inpatient and outpatient medical records, laboratory results, hospitalization records, and physician documentation throughout AZA+VEN treatment. Hematologic and non-hematologic toxicities, infectious complications, febrile neutropenia, bleeding events, tumor lysis syndrome, unplanned hospitalizations, and treatment-related mortality were specifically assessed and graded according to CTCAE version 5.0. Overall survival (OS) was defined as the interval between initiation of AZA+VEN therapy and death from any cause or last follow-up (FU). Progression-free survival (PFS) was defined as the interval between treatment initiation and documented disease progression, relapse, or death from any cause, whichever occurred first. Patients alive and progression-free at the time of analysis were censored at the date of last follow-up. Descriptive statistics were used to summarize baseline characteristics, treatment exposure, response rates, and toxicity profiles. Continuous variables were reported as medians and ranges, whereas categorical variables were summarized as frequencies and percentages. The ORR was defined as the proportion of patients achieving CR, CRi, MLFS, or PR. Survival distributions were estimated using the Kaplan–Meier method and compared using the log-rank test. Analyses of treatment response, PFS and OS in the overall cohort represented the prespecified analyses of the study. Subgroup analyses according to ELN 2022, ELN 2024, Beat-AML 2024, comorbidity indices and prior azacitidine exposure were considered exploratory and were not formally prespecified. Given the sample size and the exploratory nature of these analyses, no adjustment for multiple comparisons was applied. All reported *p*-values were two-sided, and a *p*-value < 0.05 was considered statistically significant. Statistical analyses and graphical representations were performed using Microsoft Excel (Microsoft Corporation, Redmond, WA, USA) and Python (Pandas (version 2.2.3), NumPy (version 2.3.5), Matplotlib (version 3.10.8))-based statistical packages.

## 3. Baseline Patient Characteristics, Molecular Risk Stratification, and Comorbidity Assessment

A total of 21 consecutive patients with newly diagnosed AML treated with first-line AZA+VEN were included in the study. The median age was 77.2 years (range, 66–88 years). Sixteen patients were male (76.2%) and five were female (23.8%). The median age was 76.6 years (range, 66–88 years) among males and 73 years (range, 70–84 years) among females. According to the SIE/SIES/GITMO fitness criteria, 20 patients (95.2%) were considered unfit for intensive chemotherapy, whereas one patient (4.8%) was formally fit but declined standard intensive induction therapy and was therefore treated with AZA+VEN. Regarding AML subtype, 12 patients (57.1%) had de novo AML. Secondary AML was observed in the remaining patients, including AML arising from MDS in five cases (23.8%), myeloproliferative neoplasm (MPN)-associated AML in three cases (14.3%), AML evolving from chronic myelomonocytic leukemia (CMML) in one case (4.8%), and AML developing after immune thrombocytopenia (ITP) in one case (4.8%). This case is reported separately to acknowledge the history of a prior hematologic disorder; however, no association between ITP and AML development was assumed, and the patient was therefore classified as one of the 12 patients having de novo AML. Five patients (23.8%) had previously received azacitidine for antecedent MDS before leukemic transformation, with a median of five prior AZA cycles. At baseline, the platelet count was <100,000/mm^3^ in 12 patients (57.1%) and >100,000/mm^3^ in nine patients (42.9%). Ferritin levels were ≥2500 ng/mL in one patient (4.8%). Serum albumin levels were ≥3.5 g/dL in 12 patients (57.1%), between 3.0 and 3.5 g/dL in six patients (28.6%), and <3.0 g/dL in three patients (14.3%). As previously described, these variables were collected because they constitute key components of the augmented HCT-CI. Detailed baseline characteristics are summarized in Table 1.

Conventional karyotyping identified a normal karyotype in 14 patients (66.7%), trisomy 8 in four patients (19.0%), and adverse cytogenetic abnormalities, including complex and monosomal karyotypes, in three patients (14.3%). Molecular profiling revealed *FLT3-ITD* mutations in two patients, *NPM1* mutations in two, *IDH1* mutations in two, and *IDH2* mutations in two. Myelodysplasia-related (MR) gene mutations were frequent and were detected in eight patients (38.1%), whereas *TP53* mutations were identified in two cases (9.5%), with variant allele frequencies (VAFs) of 4% and 93%, respectively. According to the ELN 2017 classification, 10 patients were categorized as adverse risk, nine as intermediate risk, and two as favorable risk. Application of the ELN 2022 criteria resulted in minimal changes, with only one patient being reclassified from adverse to intermediate risk. In contrast, implementation of the ELN 2024 classification substantially modified risk allocation, leading to the reclassification of 10 patients (47.6%) and reducing the adverse-risk group from nine to two patients. The number of favorable-risk patients increased from two to eight, mainly owing to the reclassification of cases harboring MR gene mutations. Subsequent application of the Beat AML 2024 model further redistributed patients across prognostic categories, resulting in 11 favorable-risk, six intermediate-risk, and four adverse-risk cases. Overall, 11 patients (52.4%) were reclassified between the ELN 2024 and Beat AML 2024 systems, highlighting the potential impact of emerging biologically driven prognostic models in patients receiving venetoclax-based therapy. Cytogenetic and molecular findings are summarized in Table 2.

The median ECOG performance status was 2 (range, 1–4), with 16 patients (76.2%) presenting an ECOG score ≥ 2. The median ADL, CIRS, and CCI scores were 60 (range, 45–90), 12 (range, 6–17), and 8 (range, 4–12), respectively. The median HCT-CI and augmented HCT-CI scores were 6 (range, 0–11) and 8 (range, 1–12), respectively. The median age-adjusted HCT-CI was 7 [with 9 patients <7 (42.8%), and 12 patients ≥7 (57.1%)]; however, as age adjustment represents an optional extension of the HCT-CI, the original HCT-CI was retained as the standard comorbidity measure for the main analyses. Most patients exhibited a high comorbidity burden, with 12 patients (57.1%) presenting an HCT-CI ≥ 6 and 12 patients (57.1%) an augmented HCT-CI ≥ 8. Exploratory correlation analyses showed a moderate positive association between CIRS and the augmented HCT-CI (Spearman’s ρ = 0.55, *p* = 0.010), whereas weaker correlations were observed with ECOG performance status (ρ = 0.28, *p* = 0.210), CCI (ρ = 0.28, *p* = 0.213), and ADL (ρ = 0.13, *p* = 0.585). Incorporation of serum albumin, platelet count, and ferritin into the augmented HCT-CI resulted in score redistribution in all patients. Compared with the conventional HCT-CI, the augmented HCT-CI increased by one point in seven patients (33.3%), by two points in six patients (28.6%), by three points in five patients (23.8%), and by four points in three patients (14.3%). Overall, 14 patients (66.7%) experienced an increase of at least two points, suggesting that biological parameters may substantially refine comorbidity assessment in elderly AML patients treated with AZA+VEN. Prevalent comorbidities were cardiovascular disorders (100% of patients), followed by chronic respiratory diseases (38.1%), chronic kidney disease (33.3%), diabetes mellitus (33.3%), previous malignancies (28.6%), rheumatologic disorders (23.8%), and chronic infectious conditions or multidrug-resistant bacterial colonization (47.6%). Individual patients could have more than one comorbidity; therefore, comorbidity categories should not be considered as mutually exclusive and cumulative percentages may exceed 100%. Comorbidity assessment is summarized in Table 3.

Patient-level data are presented in detail in Table 4, highlighting the substantial and frequently overlapping comorbidity burden of this cohort. Several patients had concomitant cardiovascular, respiratory, renal, metabolic, vascular, and infectious conditions, underscoring the clinical complexity and vulnerability frequently encountered in real-world AML populations requiring treatment.

### Treatment Exposure, Response, and Safety

All 21 patients received frontline AZA+VEN. The median number of treatment cycles administered was 7 (range 1–29), reflecting the heterogeneous duration of therapy observed in routine clinical practice. At the time of analysis, seven patients remained on treatment, 10 patients were alive, and 11 had died. The ORR was 57.1% (12/21 patients), comprising CR/CRi in nine patients (42.9%), MLFS in two patients (9.5%), and PR in one patient (4.8%). Among the nine patients achieving CR/CRi, 2 (22.2%) were MRD-negative. Stable disease was observed in five patients (23.8%), whereas four patients (19.0%) were classified as non-responders. Responses were generally achieved early during treatment, with a median time to response of 1.8 months and a median of two treatment cycles required to achieve CR. Among responding patients, five subsequently experienced disease relapse, with a median time to relapse of 15.6 months from treatment initiation. All these data are shown in Table 5.

Treatment adaptations were frequently required during follow-up. Azacitidine schedule modifications were performed in 12 patients (57.1%), consisting of reduction from the standard 7-day schedule to a 5-day schedule starting from cycle 2. In most cases (8/12), this adjustment was implemented after achievement of remission, whereas in four patients it was motivated by clinical considerations unrelated to response. The remaining nine patients continued treatment without azacitidine modification. Venetoclax exposure was also individualized according to hematologic recovery, infectious complications, concomitant medications, and physician judgment. Overall, 14 patients (66.7%) required venetoclax schedule modifications. The most common strategy consisted of shortening venetoclax administration to 14 days per cycle, performed in 12 patients. Specifically, seven patients received 200 mg for 14 days, three received 300 mg for 14 days, and two received 400 mg for 14 days starting from cycle 2. One patient received 400 mg for 15 days starting from cycle 1, while another received 100 mg during the first two cycles due to concomitant posaconazole administration. Venetoclax modifications were mainly driven by achievement of remission (11/14 patients), while three adjustments were performed for other clinical reasons. Importantly, most treatment modifications were implemented after achievement of response. In our clinical experience, these adaptations were not followed by an apparent increase in early relapse or disease progression and were implemented to facilitate treatment continuation while limiting prolonged interruptions. Given their response-adapted timing and the cohort size, however, their independent impact on efficacy could not be formally compared with that of unmodified treatment. Supportive care was extensively used. Granulocyte colony-stimulating factor (G-CSF) was administered to 17 patients (81.0%), with a median duration of 5.5 days. In addition, five patients underwent temporary treatment interruption (“washout”), with a median duration of 7 days, primarily to allow hematologic recovery before subsequent treatment cycles. All these data are shown in greater detail in Table 6.

Treatment was generally manageable, although hematologic toxicity was frequent, as expected in an elderly AML population receiving VEN-based therapy. All patients developed emergent cytopenia during therapy. Neutropenia was the most common hematologic adverse event, with grade 3–4 neutropenia observed in 19 patients (90.5%), including grade 4 events in 15 cases. Similarly, grade 3–4 thrombocytopenia occurred in 16 patients (76.2%), while grade 3–4 anemia was documented in 13 patients (61.9%). Despite the high incidence of severe cytopenia, these events were generally managed with supportive care measures, temporary treatment interruptions, growth factor administration, and schedule modifications, without unexpected safety signals. A total of 28 non-hematologic adverse events were recorded. Infectious complications represented the most frequent toxicity category, accounting for 14 events (14/28–50% of all non-hematologic toxicities). The most common infections involved the urinary tract (4/28–14.3%), respiratory tract (4/28–14.3%), and febrile neutropenia (2/28–7.1%). Additional infectious events included herpes zoster reactivation, influenza A infection, peripherally inserted central catheter (PICC)-related infection, and one episode of *Clostridioides difficile*-associated diarrhea. Importantly, most infectious complications were grade 2–3, and no grade 4 infectious events were observed. Gastrointestinal (GI) adverse events accounted for five events (17.9%), including nausea/vomiting and diarrhea, and were predominantly low grade. Laboratory abnormalities accounted for four events (14.3%), mainly electrolyte disturbances. Cardiovascular and respiratory toxicities were less frequent, comprising two episodes of grade 3 atrial fibrillation/flutter and one case of bronchial stricture. Overall, the safety profile observed in our cohort was consistent with that reported in pivotal clinical trials and subsequent real-world studies of AZA+VEN. Although severe hematologic toxicity was nearly universal, non-hematologic adverse events were relatively infrequent and largely manageable, supporting the feasibility of AZA+VEN even in a highly comorbid and predominantly Ferrara-unfit population. Considering the whole cohort, 6/21 patients (28.6%) experienced grade 3 infectious events; however, none of these infections was considered life-threatening or associated with clinically significant organ dysfunction. No grade 4 infectious events were observed. Febrile neutropenia occurred in 2/21 patients (9.5%). Two patients (9.5%) required unplanned hospitalization during treatment: one for acute renal failure, and one for bleeding from a colonic telangiectasia followed by *Clostridioides difficile* infection. Both the acute renal failure and gastrointestinal bleeding were considered unrelated to AZA+VEN. No cases of tumor lysis syndrome were observed, and no deaths were considered directly attributable to treatment. Treatment-related toxicities and additional safety outcomes are summarized in Table 7.

## 4. Results

**Figure 2:**
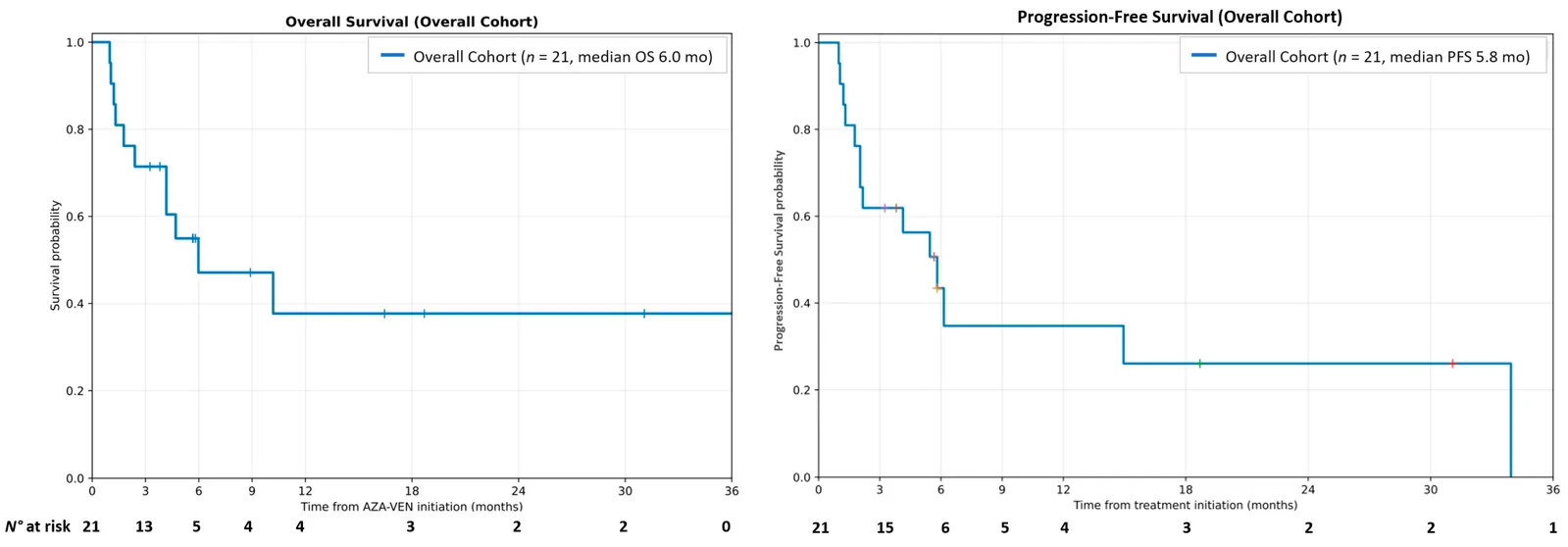
Kaplan–Meier estimates of overall survival (OS, **left**) and progression-free survival (PFS, **right**) in the overall cohort treated with AZA+VEN. Median PFS was 5.8 months, and median OS was 6.0 months (95% CI, 2.4 months–NR).

At the data cutoff (31 May 2026), 10/21 patients (47.6%) were alive and censored at their last follow-up, whereas 11/21 (52.4%) had died. Among the 11 deaths, 5/11 (45.5%) were related to progressive/refractory AML, whereas 6/11 (54.5%) occurred from other causes. Among the latter six patients, deaths were associated with infectious/respiratory complications (n = 2), cardiovascular complications (n = 1), multiorgan failure (n = 1), and multifactorial clinical deterioration (n = 2), including infection, cachexia, and cardiovascular comorbidity. Thirty- and 60-day mortality rates were 4.8% (1/21) and 23.8% (5/21), respectively. No deaths were attributed to acute or direct treatment toxicity as the primary cause of death. However, particularly in patients with progressive disease, the terminal clinical course was often multifactorial and treatment-related myelosuppression may have contributed to infectious or other complications alongside AML progression and underlying comorbidities. Considering the limited number of cause-specific events, causes of death were summarized descriptively rather than analyzed using a competing-risk approach. Given the cohort size and number of events, multivariable analyses were not performed and the results were primarily interpreted within an exploratory framework. Median follow-up was calculated using the reverse Kaplan–Meier method and was 15.4 months. Kaplan–Meier analysis was performed in the overall cohort (n = 21). Median progression-free survival (PFS) was 5.8 months. The PFS curve showed a progressive decline during follow-up, with estimated PFS probabilities of approximately 35% at 6 months and 26% beyond 12 months. Median overall survival (OS) was 6.0 months. The OS curve demonstrated an initial reduction in survival probability during the early months of follow-up, followed by a relatively stable plateau, with an estimated survival probability of approximately 38% maintained from month 10 onward (Figure 2).

**Figure 3:**
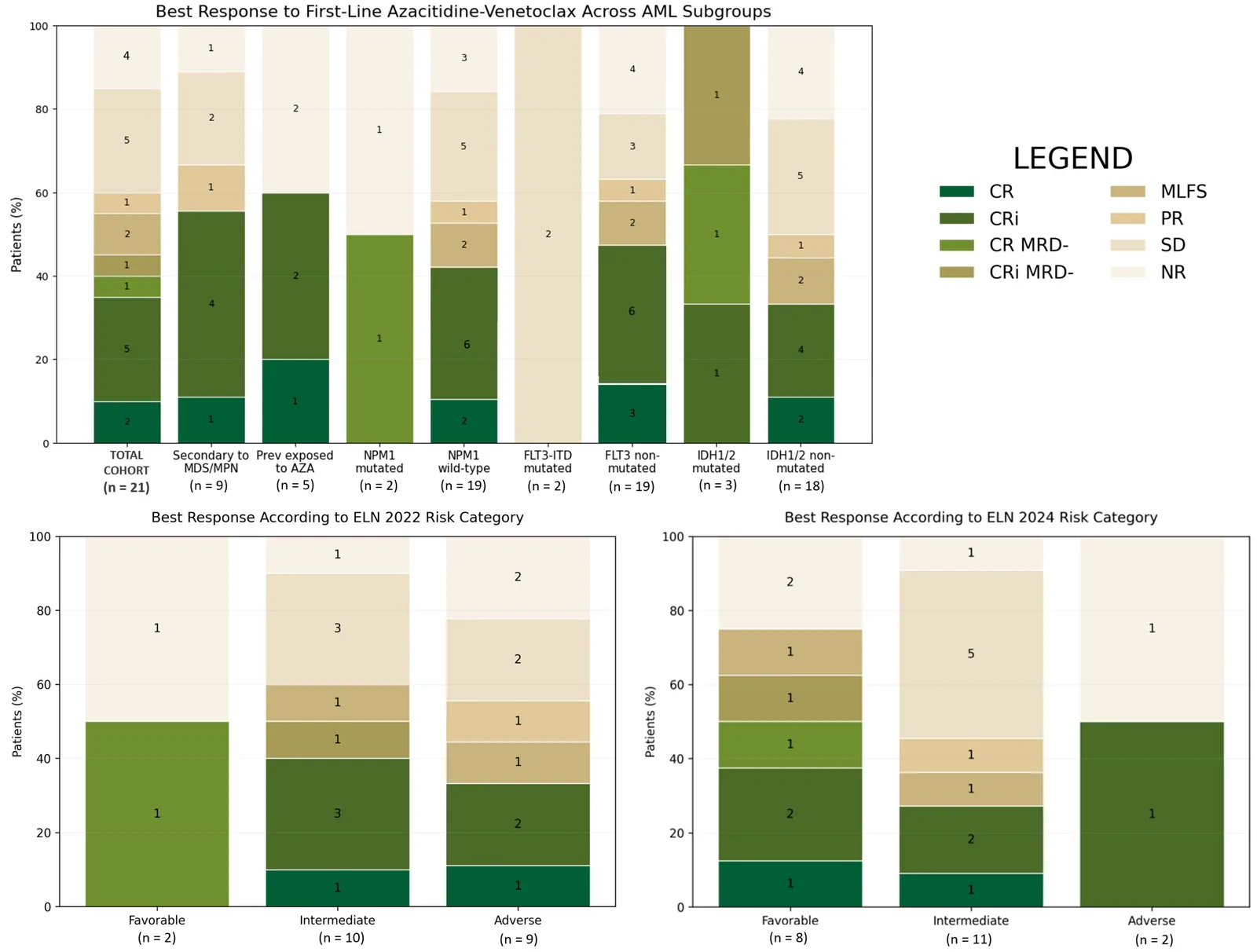
Best response to first-line AZA+VEN according to clinical, molecular, and ELN risk subgroups. Stacked bar charts show the distribution of best response categories among patients with AML treated with first-line AZA+VEN. The upper panel illustrates response rates according to baseline clinical and molecular characteristics. The lower panels display response distributions according to the ELN 2022 and ELN 2024 risk classifications. Numbers within each bar indicate the absolute number of patients in each response category, while bar heights represent percentages within each subgroup. Response categories include complete remission (CR), complete remission with incomplete hematologic recovery (CRi), morphologic leukemia-free state (MLFS), partial remission (PR), stable disease (SD), and no response (NR). Measurable residual disease (MRD) negativity should be considered a qualifier of CR/CRi rather than a separate response category and is highlighted by a distinct color to provide additional information on molecular response depth. Abbreviations: AML (acute myeloid leukemia); MDS (myelodysplastic syndrome); MPN (myeloproliferative neoplasm); ELN (European LeukemiaNet).

Using the ELN 2022 criteria, the favorable-risk group comprised only two patients, of whom one achieved CR with MRD negativity (50%) and one was non-responsive (NR, 50%). Among the 10 patients classified as intermediate risk, the composite complete remission rate (CR/CRi, including MRD-negative responses) was 60% (6/10), consisting of one CR (10%), three CRi (30%), one MRD-negative CRi (10%; *IDH1* mutation not detectable), and one MRD-negative CR (10%; both *NPM1* and *IDH2* not detectable). Additional responses included one MLFS (10%), while three patients achieved stable disease (30%). In contrast, the adverse-risk group (n = 9) showed a lower composite CR/CRi rate of 33.3% (3/9), including one CR (11.1%) and two CRi (22.2%), whereas non-response or disease stabilization was observed in six patients (66.7%), including two NR (22.2%), two SD (22.2%), one PR (11.1%), and one MLFS (11.1%). Overall, the ELN 2022 classification showed a trend toward higher remission rates in favorable- and intermediate-risk patients (Figure 2). Application of the ELN 2024 criteria substantially redistributed patients across risk groups and resulted in a more balanced distribution of clinical outcomes. Among the eight patients classified as favorable risk, 5/8 (62.5%) achieved CR/CRi, including 2/8 CR (25.0%) and 3/8 CRi (37.5%); two of these five responders were also MRD-negative. One patient achieved MLFS (12.5%), while two patients were non-responders (25%). The intermediate-risk group comprised 11 patients and demonstrated a composite CR/CRi rate of 36.4% (4/11), including two CRi (18.2%) and two CR (18.2%), together with one PR (9.1%), one MLFS (9.1%), and five patients with stable disease or non-response (45.5%). Notably, the adverse-risk group was reduced to only two patients, one of whom achieved CRi (50%) while the other was non-responsive (50%) (Figure 2). Despite the limited number of cases, ELN 2024 appeared to enrich the favorable-risk category for responders by reclassifying several patients who would have been considered adverse risk according to ELN 2022. When responses were analyzed independently of ELN classification, the ORR was 57.1% (12/21), comprising CR/CRi in nine patients (42.9%), MLFS in two patients (9.5%) and PR in one patient (4.8%). Among the nine patients achieving CR/CRi, 2 (22.2%) were MRD-negative. Stable disease was observed in five patients (23.8%), while four patients (19.0%) had no response (Figure 3).

**Figure 4:**
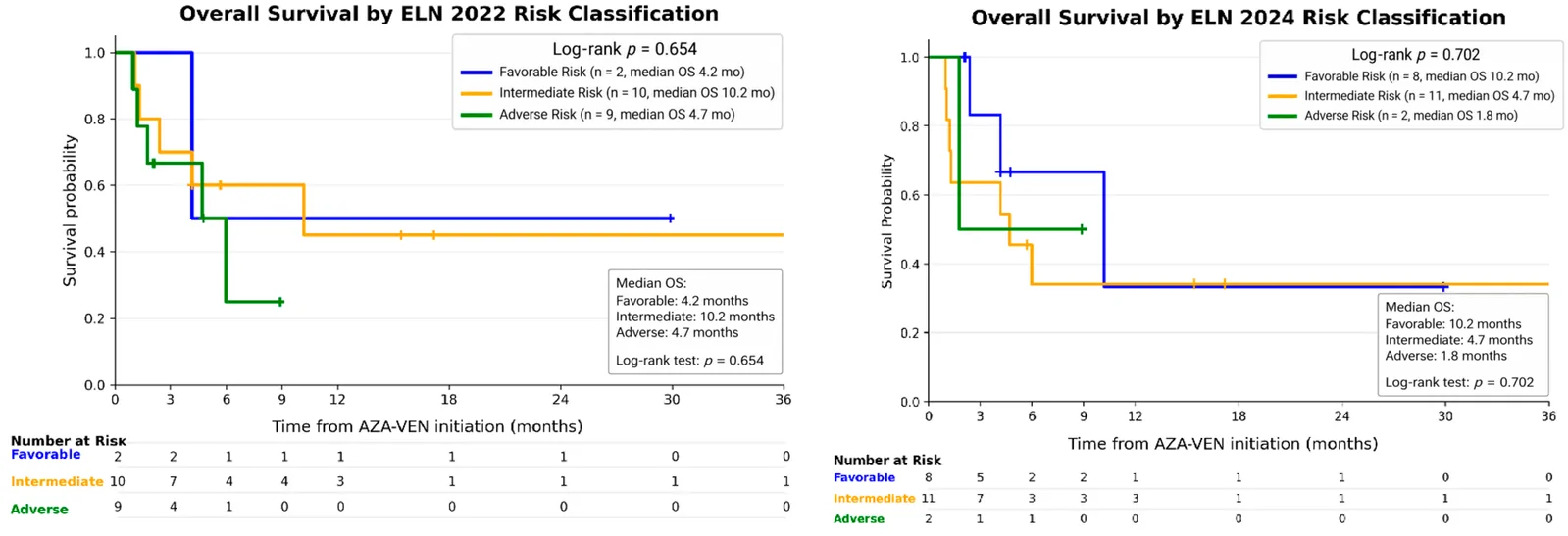
Kaplan–Meier OS according to ELN 2022 (**left**) and ELN 2024 (**right**) risk classifications in patients treated with AZA+VEN. According to ELN 2022 risk classification, Median OS was 4.2 months (95% CI, 4.2–NR) in the favorable-risk group (n = 2), 10.2 months (95% CI, 1.1–NR) in the intermediate-risk group (n = 10), and 4.7 months (95% CI, 1.0–NR) in the adverse-risk group (n = 9) (log-rank *p* = 0.654). According to ELN 2024 risk classification, median OS was 10.2 months (95% CI, 2.4 months–NR) in the favorable-risk group (n = 8), 4.7 months (95% CI, 1.1 months–NR) in the intermediate-risk group (n = 11), and 1.8 months (95% CI, 1.8 months–NR) in the adverse-risk group (n = 2). Numbers at risk are shown below the Kaplan–Meier curves.

Using the ELN 2022 criteria, median OS was 4.2 months (95% CI, 4.2 months–NR) in favorable-risk patients (n = 2), 10.2 months (95% CI, 1.1 months–NR) in intermediate-risk patients (n = 10), and 4.7 months (95% CI, 1.0 months–NR) in adverse-risk patients (n = 9), with no significant differences between groups (log-rank *p* = 0.654). Moreover, substantial overlap in survival was observed between the ELN 2022 intermediate- and adverse-risk groups in this cohort of elderly and medically unfit patients treated with AZA+VEN. Application of the ELN 2024 classification resulted in a substantial redistribution of patients across risk categories and modified the survival pattern observed with ELN 2022. The favorable-risk group increased from two to eight patients and showed a median OS of 10.2 months (95% CI, 2.4 months–NR), whereas the intermediate-risk group (n = 11) had a median OS of 4.7 months (95% CI, 1.1 months–NR). The adverse-risk category was reduced to only two patients, with a median OS of 1.8 months (95% CI, 1.8 months–NR). When considered together with response data, ELN 2024 appeared to provide a clinically meaningful redistribution of patients, with a closer correspondence between risk classification and survival outcomes. Several patients who achieved complete remission were reclassified from intermediate or adverse categories into the favorable-risk group, resulting in an enrichment of both response and survival outcomes rates and OS within this category. Overall survival according to ELN 2022 and ELN 2024 risk stratification is shown in Figure 4.

**Figure 5:**
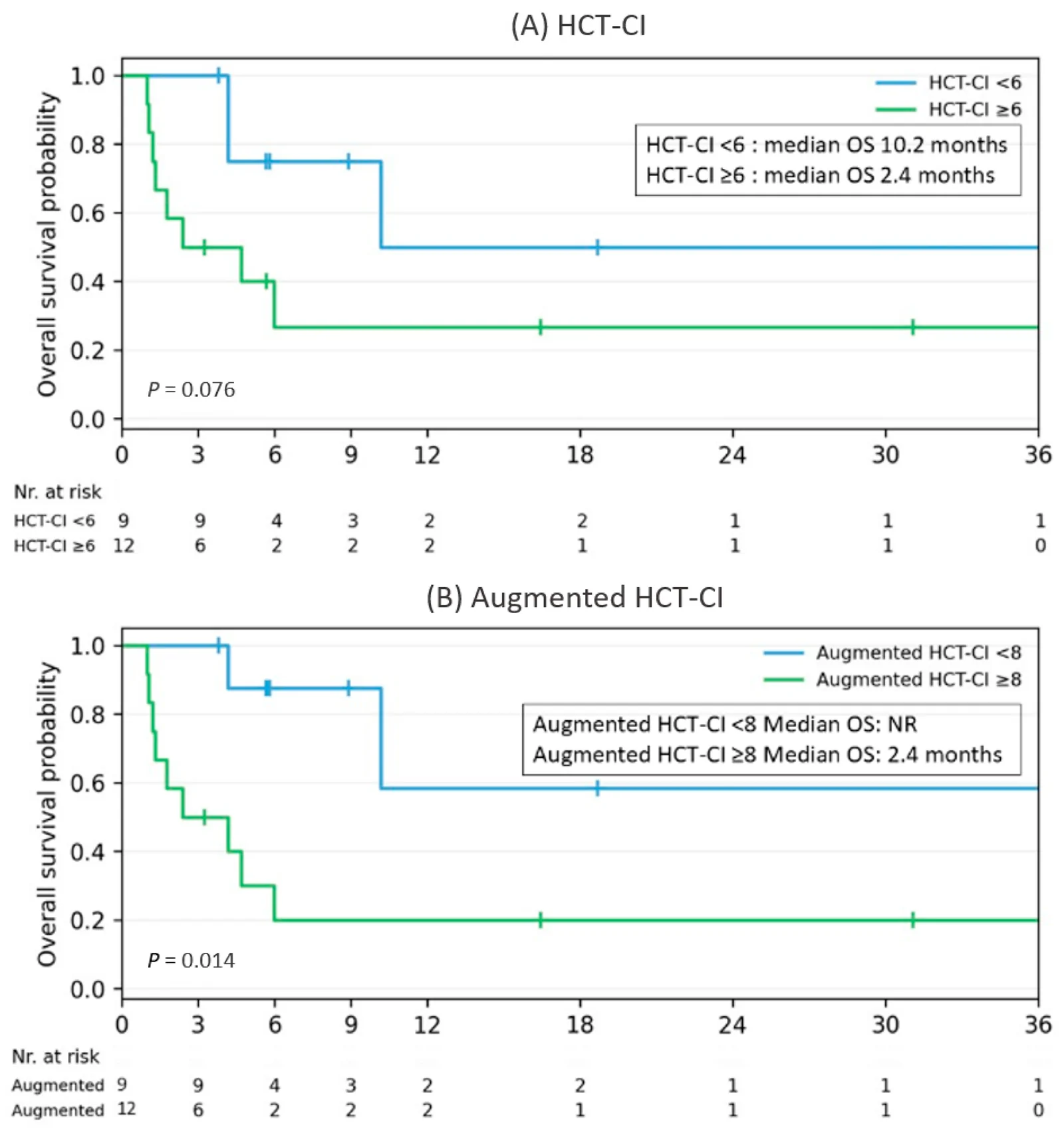
Kaplan–Meier overall survival (OS) according to baseline comorbidity burden assessed by the HCT-CI (**A**) and augmented HCT-CI (**B**). Patients with lower comorbidity scores showed longer survival compared with those with higher scores. According to HCT-CI, median OS was 10.2 months (95% CI, 4.2 months–NR) in patients with HCT-CI < 6 (n = 9) and 2.4 months (95% CI, 1.1 months–NR) in those with HCT-CI ≥ 6 (n = 12) (log-rank *p* = 0.076). According to augmented HCT-CI, median OS was not reached (95% CI, 4.2 months–NR) in patients with an augmented HCT-CI < 8 (n = 9), whereas patients with an augmented HCT-CI ≥ 8 (n = 12) had a median OS of 2.4 months (95% CI, 1.1–6.0 months) (log-rank *p* = 0.014). Overall survival according to augmented HCT-CI. Median OS was not reached (95% CI, 4.2 months–NR) in patients with an augmented HCT-CI < 8 (n = 9), whereas patients with an augmented HCT-CI ≥ 8 (n = 12) had a median OS of 2.4 months (95% CI, 1.1–6.0 months) (log-rank *p* = 0.014). Numbers at risk are shown below the Kaplan–Meier curves. Abbreviations: OS (overall survival); HCT-CI (Hematopoietic Cell Transplantation–Comorbidity Index); FU (follow-up); NR (not reached).

According to the Beat AML 2024 classification, 11 patients (52.4%) were categorized as favorable risk, six (28.6%) as intermediate risk, and four (19.0%) as adverse risk. Composite complete remissions (CR/CRi ± MRD negativity) were observed in 54.5% of favorable-risk patients, compared with 33.3% and 25.0% in the intermediate- and adverse-risk groups, respectively. Stable disease was the most frequent outcome among adverse-risk patients (50.0%), whereas response patterns in the intermediate-risk category were markedly heterogeneous. To investigate the prognostic impact of baseline comorbidity burden, patients were stratified according to the median values of the HCT-CI and augmented HCT-CI scores (Table 3). The median value was selected as the cutoff because it provided a balanced distribution of patients between groups, minimizing disparities in sample size and allowing a more robust comparison of survival outcomes. Using a cutoff of six points, patients with an HCT-CI < 6 (n = 9) had a median OS of 10.2 months (95% CI, 4.2 months–NR), compared with 2.4 months (95% CI, 1.1 months–NR) in those with an HCT-CI ≥ 6 (n = 12), with a trend toward improved survival in the lower-score group (log-rank *p* = 0.076). A separation of the survival curves was observed (log-rank *p* = 0.076), suggesting a clinically relevant association between increasing comorbidity burden and inferior outcomes. When the augmented HCT-CI was applied, using a median cutoff of eight points, prognostic discrimination became more pronounced. Patients with an augmented HCT-CI < 8 (n = 9) had a median OS that was not reached (95% CI, 4.2 months–NR), whereas those with augmented HCT-CI ≥ 8 (n = 12) had a median OS of 2.4 months (95% CI, 1.1–6.0 months) (log-rank *p* = 0.014) (Figure 5). Given the small sample size and absence of formal comparative model-performance analyses, this finding should be interpreted as an exploratory association rather than evidence of superiority over the conventional HCT-CI or ELN-based risk classifications.

**Figure 6:**
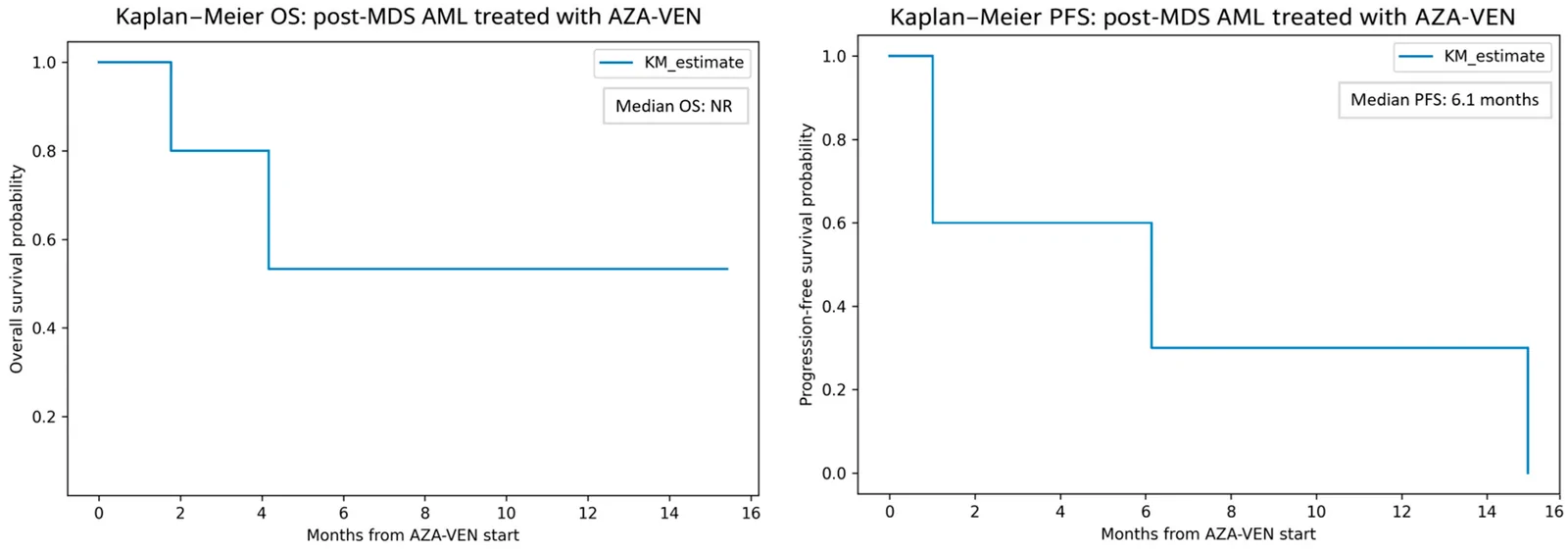
Kaplan–Meier estimates of overall survival (OS) and progression-free survival (PFS) in post-MDS AML patients treated with AZA+VEN.

A subset of five patients in the present cohort had AML evolving from previously treated MDS and had already received azacitidine before leukemic transformation (median, five cycles). Despite prior exposure to hypomethylating agents, clinical benefit was observed in three of five patients treated with AZA+VEN. Median OS was not reached, while median PFS was 6.1 months. Although two patients were primarily refractory and experienced rapid disease progression, three patients derived clinical benefit from AZA+VEN. Notably, one patient with therapy-related MDS/AML and adverse molecular features achieved rapid hematologic improvement, with restoration of effective erythropoiesis and transfusion independence after only two treatment cycles, reaching normal hemoglobin and platelet values (Hb 14 g/dL, platelets > 150,000/mm^3^). Another patient maintained disease control for more than one year (14 cycles), whereas a third patient, despite harboring a TP53 mutation, completed six treatment cycles and achieved transfusion independence for three consecutive cycles (cycles 2–4). The individual clinical, molecular, and treatment characteristics of these patients are summarized in Table 8, while Kaplan–Meier estimates of OS and PFS are shown in Figure 6. Although the small number of patients precludes definitive conclusions, these data are clinically relevant, as post-MDS AML following azacitidine exposure represents a challenging therapeutic setting. The clinical benefit described in three of five patients suggests that prior HMA exposure does not invariably preclude response to AZA+VEN. This observation should be regarded as hypothesis-generating and warrants further investigation across broader patient populations.

## 5. Discussion

The introduction of venetoclax in combination with HMAs has profoundly changed the therapeutic landscape of older and medically unfit patients with AML, establishing AZA+VEN as the current standard of care (SOC) following the pivotal VIALE-A study and its long-term follow-up analyses [3,23]. However, the optimal venetoclax schedule and duration during remission remain undefined. Venetoclax dosing must balance efficacy against toxicity, particularly neutropenia, which is a key determinant of infection-related treatment risk [24]. Real-world studies provide an essential complement to prospective trials and allow assessment of treatment effectiveness in populations often underrepresented in clinical research. The present study confirms the substantial clinical activity of frontline AZA+VEN in a cohort characterized by advanced age, high comorbidity burden, and a large proportion of secondary AML. It should be noted that a history of antecedent MDS or MPN and prior treatment with azacitidine represents exclusion criteria of the VIALE-A study [25]; therefore, evidence from real-world practice is essential to further define the true potential of this therapeutic strategy in previously underrepresented patient populations. The ORR and composite CR/CRi rate observed in our series compare favorably with published real-world experiences and support the reproducibility of venetoclax-based outcomes outside clinical trials [26,27]. Responses were generally achieved rapidly, with a median time to response below 2 months, consistent with previous observations showing that venetoclax induces early cytoreduction and prompt hematopoietic recovery in responding patients [28]. Importantly, 16.7% of responding patients (2/12) achieved molecular MRD-negative remissions (i.e., undetectable *NPM1*, *IDH1*, or *IDH2* mutations), a finding of relevance given the growing evidence supporting MRD negativity as one of the strongest predictors of long-term outcome in AML treated with venetoclax-containing regimens [29,30]. *FLT3-ITD*-mutated patients (2/21–9.5%) did not achieve a significant response and progressed after a few cycles, partly due to comorbidities that prevented continuous treatment administration without interruptions or dose adjustments. Studies have shown that venetoclax can achieve meaningful responses in *FLT3*-mutated unfit patients (particularly in those not eligible for *FLT3* inhibitors in first line), although triplet regimens have demonstrated superior outcomes [31]. We also compared ELN 2022 and ELN 2024 classifications. The ELN 2022 recommendations were largely derived from cohorts receiving intensive chemotherapy and classify AML harboring myelodysplasia-related (MR) mutations as adverse risk [13]. However, emerging evidence indicates that several MR-mutated AML subgroups derive substantial benefit from venetoclax-based therapy, challenging prognostic assumptions established in the intensive-treatment era [32,33,34]. Accordingly, the recently proposed ELN 2024 less-intensive classification reclassified nearly half of our patients, markedly reducing the adverse-risk category and enriching the favorable-risk group. Notably, patients reassigned to favorable risk showed higher remission rates and longer survival, although the OS comparison across ELN 2024 risk groups did not reach statistical significance and the adverse-risk group included only two patients. However, when considered together with response data, ELN 2024 therefore appeared to provide a clinically meaningful redistribution of patients, with a closer correspondence between risk classification, remission rates and survival outcomes. These findings align with emerging evidence suggesting that ELN 2024 may better capture disease characteristics associated with response to HMA+VEN therapy [9,14]. An additional strength of our analysis is the comprehensive assessment of patient fitness. While molecular classification remains fundamental, increasing evidence suggests that biological age and frailty may influence outcomes as much as leukemia genetics in older AML patients [35]. The HCT-CI, originally developed by Sorror et al., is one of the most widely adopted tools for quantifying comorbidity burden in patients undergoing allogeneic hematopoietic stem cell transplantation (allo-HSCT) [10]. By systematically incorporating pre-existing organ dysfunction and relevant comorbidities, the score was designed to predict non-relapse mortality (NRM) and overall survival (OS) after transplantation and has since been validated in multiple independent cohorts. Vaughn et al. subsequently developed the augmented HCT-CI, integrating three readily available laboratory biomarkers [serum albumin, ferritin, and PLT count] into the original score [11]. This modified model demonstrated improved prognostic discrimination compared with the conventional HCT-CI, highlighting the added value of biomarkers reflecting systemic inflammation, iron overload, and residual hematopoietic reserve [36]. Although initially conceived for transplant candidates, interest in the HCT-CI and augmented HCT-CI has progressively expanded beyond the transplant setting, particularly in older patients with AML receiving lower-intensity therapies. While disease-related characteristics (molecular and cytogenetic) remain central to prognostic assessment, our study further supports the growing concept that, in elderly and medically unfit AML populations, comprehensive evaluation of comorbidities and fitness provides a more holistic view of the patient and supports individualized therapeutic strategies to ensure treatment adherence. Patients with lower augmented HCT-CI scores experienced longer survival, whereas those with higher scores showed earlier mortality. The separation of the Kaplan–Meier curves occurred early after treatment initiation and remained evident throughout follow-up, suggesting a potentially relevant association between the clinical and biological factors captured by the augmented HCT-CI and survival. In our cohort, higher augmented HCT-CI scores were associated with shorter OS, with an early separation of the Kaplan–Meier curves that persisted during follow-up. Although the conventional HCT-CI showed a similar trend, the association was more pronounced when the augmented HCT-CI was applied. Importantly, augmented HCT-CI should not be regarded exclusively as a measure of comorbidity. By incorporating serum albumin, platelet count and ferritin, the score may also capture nutritional and inflammatory status, hematopoietic reserve, and disease-related factors. Its association with survival may therefore reflect a broader, integrated host–disease fitness profile, potentially relevant to outcome assessment in older and unfit patients receiving AZA+VEN. Notably, the augmented HCT-CI distinguished patient groups with different survival outcomes in our cohort, supporting further investigation of its potential prognostic relevance in elderly and medically unfit patients receiving this therapeutic regimen. The association between higher augmented HCT-CI scores and shorter OS suggests that the incorporation of laboratory parameters may provide additional information beyond conventional comorbidity assessment. However, given the exploratory nature of the analysis, the limited cohort size and the absence of external validation, these findings should not be interpreted as demonstrating formal prognostic superiority over the conventional HCT-CI. Rather, they represent a clinically relevant, hypothesis-generating signal that warrants further evaluation in larger independent cohorts and, ideally, prospective studies. No multivariable analyses were performed because the cohort size did not permit reliable model adjustment without substantial risk of overfitting. Overall, our findings support continued investigation of the augmented HCT-CI as a readily applicable approach to baseline risk assessment that integrates conventional comorbidity burden with additional laboratory parameters. Further prospective evaluation will be required to determine whether this integrated assessment may ultimately complement established clinical and disease-related risk factors in patients receiving non-intensive VEN-based AML therapy.

Treatment modifications were frequently required in routine clinical practice. More than half of the patients required azacitidine schedule modifications, and two-thirds underwent venetoclax dose reductions, most frequently shortening treatment to 14 days per cycle. Similar patterns have been reported in multiple real-world cohorts and increasingly reflect routine clinical practice [37]. Importantly, treatment modifications were frequently implemented during therapy, particularly after achievement of response, reflecting individualized real-world management of AZA+VEN treatment according to hematologic recovery, treatment-related toxicity, comorbidities and overall tolerability. In this clinical context, schedule and dose adaptations were primarily aimed at limiting cumulative myelosuppression and facilitating treatment continuation. However, as most modifications were introduced after response had already been achieved, comparisons according to subsequent treatment adaptation would be inherently affected by time-dependent selection bias. Treatment modifications were therefore interpreted descriptively in relation to their timing and clinical context, without attributing subsequent efficacy outcomes to shortened venetoclax exposure or azacitidine schedule modification. The safety profile observed in our cohort was consistent with previous reports, with hematologic toxicity representing the most frequent adverse event and infectious complications remaining largely manageable through supportive care, growth factor support, and individualized treatment scheduling [38]. Finally, our study provides observations regarding AML evolving from antecedent MDS previously exposed to azacitidine [39]. Although this population is generally considered difficult to treat, three of five patients derived meaningful benefit from AZA+VEN, including durable disease control and restoration of transfusion independence. The median number of prior azacitidine cycles was five, with no or transient hematologic responses. After venetoclax initiation, median time to CR/MLFS was 1.7 months. Venetoclax was administered for 14 days per cycle. One patient completed six cycles, achieving transient transfusion independence despite *TP53* mutation and monosomal karyotype. A second patient completed 14 cycles. A third patient (ELN 2024 adverse risk) achieved transfusion independence (Hb 14 g/dL, platelets > 150,000/mm^3^) and complete hematologic response after only two cycles. A fourth patient, harboring *TP53* mutation and complex karyotype, discontinued treatment after two cycles due to clinical deterioration. Although emerging evidence suggests that *TP53*-mutated AML associated with additional MR mutations may have relatively better outcomes [40], this category still presents dismal outcome and further studies are needed to improve OS in this challenging setting. Evidence specifically addressing post-MDS AML still remains limited. The clinical benefit observed in three of five patients with post-MDS AML previously exposed to azacitidine suggests that prior HMA exposure does not invariably preclude response to AZA+VEN. However, given the small subgroup size, this observation should be interpreted cautiously and warrants further investigation in broader patient populations. This study has some limitations, primarily related to its retrospective, single-center design and relatively small sample size (N = 21). Accordingly, analyses according to ELN risk category, Beat AML classification, prior azacitidine exposure, HCT-CI, and augmented HCT-CI should be interpreted as exploratory rather than as formal comparisons of prognostic performance. Similarly, the cutoffs applied in the comorbidity analyses warrant validation in independent cohorts. Nevertheless, the observed associations provide clinically relevant hypothesis-generating evidence that may inform future studies and should be further investigated in larger, preferably multicenter cohorts.

## 6. Conclusions

Venetoclax combined with hypomethylating agents has substantially expanded therapeutic opportunities for older and medically unfit patients with AML, a population often underrepresented in clinical trials because of age, comorbidities, and impaired functional status. In this real-world study, we evaluated frontline AZA+VEN by considering molecular risk classification, comorbidity burden, treatment adaptations, and previous HMA exposure. Our findings highlight the clinical relevance of ELN 2024 risk stratification in this setting, while also emphasizing the contribution of patient fitness and comorbidities to outcome. Of particular interest, the association between augmented HCT-CI and survival suggests that combining conventional comorbidity assessment with laboratory parameters reflecting broader host–disease fitness may add clinically relevant prognostic information. Taken together, these observations favor a more integrated assessment of leukemia-related risk and patient-related vulnerability when evaluating older and unfit patients treated with frontline AZA+VEN. Confirmation in independent patient series is warranted.

## Figures and Tables

**Table 1:** Baseline patient characteristics.

**Patients**	**N° = 21**	**%**
M	16/21	76.2%
F	5/21	23.8%
**Age, median (range)**	**77.2 (66–88)**	
M F	76.6 (66–88) 73 (70–84)	-
**Leukemia type**	***N°***	
De novo	12/21	57.1%
Secondary to MDS	5/21	23.8%
Secondary to MPN or MDS/MPN Therapy-related (prior CHT/RT for unrelated cancer) Relapsed/Refractory (at baseline)	3/21 (2 MFI; 1 CMML) 1/21 0/21	14.3% 4.8% 0%
**Prior AZA for MDS** Cycles, median (range)	***N°* = 5/21** 5 (1–8)	**23.8%**
**PLT**	***N°*** **of patients**	
<100.000/mm^3^	12/21	57.1%
≥100.000/mm^3^	9/21	42.9%
**Ferritin**	***N°*** **of patients**	
≥2500 ng/mL	1/21	4.8%
<2500 ng/mL	20/21	95.2%
**Albumin**	***N°*** **of patients**	
≥3.5	12/21	57.1%
3–3.5	6/21	28.6%
<3	3/21	14.3%
**Ferrara criteria**	***N°***	
**Unfit for CHT** **fit**	20/21 1/21	95.2% 4.8%

Abbreviations: M (male); F (female); MDS (myelodysplastic syndrome); MPN (myeloproliferative neoplasm); CMML (chronic myelomonocytic leukemia); MFI (myelofibrosis); CHT (chemotherapy); RT (radiotherapy); AZA (azacitidine); PLT (platelet count); CHT (chemotherapy). Data are reported as n (%) unless otherwise specified. Bold text identifies the main variable categories in the table. Continuous variables are expressed as median (range).

**Table 2:** Cytogenetic and molecular characteristics with risk stratification according to ELN 2017, ELN 2022, ELN 2024, and Beat AML 2024 classifications.

PT	Karyotype	Mutations (Standard Sequencing)	Mutations (NGS)	ELN 2017	ELN 2022	ELN 2024	BEAT AML 2024
1	46 XX	FLT3 ITD	DNMT3A	ADV	INT	INT	FAV
2	46 XY	FLT3 ITD	WT1	ADV	INT	INT	INT
3	46 XY	no	SRSF2; ASXL1; RUNX1	ADV	ADV	FAV	INT
4	46 XX	no	U2AF1	INT	INT	FAV	FAV
5	Complex Monosomal	no	TP53 c.742C>T p. VAF:43%	ADV	ADV	ADV	ADV
6	46 XY	no	no	INT	INT	INT	FAV
7	46 XY	no	no	INT	INT	INT	FAV
8	Complex	no	TP53 c.842A>G p VAF:93%	ADV	ADV	ADV	ADV
9	47, XX, +8[16]	no	RUNX1; EZH2; PTPN11; NRAS; KRAS	ADV	ADV	INT	ADV
10	46 XY	NPM1	no	FAV	FAV	FAV	FAV
11	46 XY	NPM1; IDH2	no	FAV	FAV	FAV	FAV
12	47, XY, +8[8]/46, XY[2]	IDH1	WT1	INT	INT	FAV	FAV
13	46 XY	no	ZRSR2; RUNX1; EZH2	ADV	ADV	FAV	INT
14	46 XY	no	ASXL1; ZRSR2; ASXL1	ADV	ADV	FAV	INT
15	46 XY	no	no	INT	INT	INT	FAV
16	46 XY	no	no	INT	INT	INT	FAV
17	46 XY	no	no	INT	INT	INT	FAV
18	45, XX, −7	no	NRAS; ASXL1	ADV	ADV	INT	INT
19	47, XY, +8	IDH2	U2AF1; MPL	INT	ADV	INT	INT
20	47, XX, +8[4]/46, XX[16]	IDH1	no	INT	INT	FAV	FAV
21	46 XY	no	SRSF2; RUNX1; STAG2; KRAS	ADV	ADV	INT	ADV
-	14 NORMAL 4 Trisomy 8 3 ADVERSE	14 NO 7 YES	8 NO 8 MR mutations 2 TP 53 mut	**10 ADV** 9 INT 2 FAV	**9 ADV** 10 INT 2 FAV	2 ADV 11 INT **8 FAV**	4 ADV 6 INT **11 FAV**

Abbreviations: FAV (favorable); INT (intermediate), ADV (adverse); AML (acute myeloid leukemia); ELN (European LeukemiaNet).

**Table 3:** Comorbidity burden, functional status, and major comorbid conditions of AML patients treated with frontline AZA+VEN.

Comorbidity Scores	*N°*	%
**ADL, median (range)**	**60 (45–90)**	
Pts < 60	9/21	42.9%
Pts ≥ 60	12/21	57.1%
**CIRS, median (range)**	**12 (6–17)**	
Pts < 12	11/21	52.4%
Pts ≥ 12	10/21	47.6%
**CCI, median (range)**	**8 (4–12)**	
Pts < 8	9/21	42.9%
Pts ≥ 8	12/21	57.1%
**Median ECOG-PS**	**2 both M/F (1–4)**	
Pts < 2	5/21	23.8%
Pts ≥ 2	16/21	76.2%
**HCT-CI, median (range)**	**6 (0–11)**	
Pts < 6	9/21	42.9%
Pts ≥ 6	12/21	57.1%
**Age-adjusted HCT-CI, median (range)**	**7 (1–12)**	
Pts < 7	9/21	42.9%
Pts ≥ 7	12/21	57.1%
**Augmented HCT-CI, median (range)**	**8 (1–12)**	
Pts < 8	9/21	42.9%
Pts ≥ 8	12/21	57.1%
**COMORBIDITIES**	***N°***	**%**
**Cardiac**	21/21	100%
- CIHD	3/21	14.3%
- AF	3/21	14.3%
- HHD	12/21	57.1%
- Mild-moderate valvular disease	15/21	71.4%
- Others (extrasystoles)	2/21	9.5%
**Pulmonary**		
- COPD	8/21	38.1%
- Others (increased bronchial markings)	2/21	9.5%
**Renal**		
- CKD II	3/21	14.3%
- CKD IIIA	3/21	14.3%
- CKD IIIB	1/21	4.8%
**Vascular**		
- PAD	2/21	9.5%
- Atheromasia	2/21	9.5%
- Previous TIA	1/21	4.8%
**Endocrine/Metabolic**		
- Hypothyroidism	4/21	19.0%
- DM type II	7/21	33.3%
- Dyslipidemia	3/21	14.3%
- Hepatic steatosis/fibrosis	2/21	9.5%
**Rheumatologic**		
- Arthrosis/arthritis	5/21	23.8%
**Previous Cancer**		
- Not CHT/RT	5/21	23.8%
- CHT+RT (colon)	1/21	4.8%
**Infective**		
- CRE positivity (rectal swab)	7/21	33.3%
- Recent urinary infection	2/21	9.5%
- Latent TB in prophylaxis	1/21	4.8%

Baseline fitness and comorbidity assessment of the study cohort. Bold text identifies the main variable categories in the table. Data are presented as median (range) or *n/N* (%), as appropriate. Individual patients could have more than one comorbidity; therefore, comorbidity categories are not mutually exclusive and cumulative percentages may exceed 100%. Abbreviations: ADL (Activities of Daily Living); CIRS (Cumulative Illness Rating Scale); CCI (Charlson Comorbidity Index); ECOG-PS (Eastern Cooperative Oncology Group Performance Status); HCT-CI (Hematopoietic Cell Transplantation–Comorbidity Index); CIHD (chronic ischemic heart disease); AF (atrial fibrillation); HHD (hypertensive heart disease); COPD (chronic obstructive pulmonary disease); CKD (chronic kidney disease); PAD (peripheral artery disease); TIA (transient ischemic attack); DM (diabetes mellitus); CHT (chemotherapy); RT (radiotherapy); CRE (carbapenem-resistant enterobacterales); TB (tuberculosis).

**Table 4:** Individual patient-level functional status, comorbidity scores, and baseline comorbidity profile.

Patient	ADL	CIRS	CCI	ECOG	HCT-CI	Augmented HCT-CI	COMORBIDITIES
1	50	15	9	2	6	8	CIHD, AH, valvular disease; CKD IIIA; cerebral vasculopathy; HCV; CRE rectal colonization
2	65	10	9	1	2	3	AH, mild valvular disease; CKD II; PAD; COPD
3	55	12	7	2	2	5	AH, mild valvular disease; COPD; hypothyroidism; aortic atherosclerosis
4	90	7	8	1	7	10	Valvular disease; bronchiectasis/obstructive bronchiolitis; pulmonary cavitation; CRE rectal colonization
5	65	11	8	2	0	4	AH, supraventricular extrasystoles; CKD IIIA; spondyloarthrosis; latent TB
6	60	11	6	2	3	6	Hypertensive heart disease, AV block; previous prostate cancer
7	65	14	10	2	7	9	DM II, dyslipidemia; AH/aortic stenosis; CKD; anxiety-depressive disorder; CRE rectal colonization
8	65	16	8	2	7	11	AH, CIHD; COPD; DM II; CKD
9	55	12	8	2	6	9	AF, multivalvular disease; recent KPC urinary infection
10	55	13	9	3	5	9	CKD IIIB; pleural effusion; multivalvular disease; spondyloarthrosis; fragility fractures; CRE rectal colonization
11	60	16	7	1	8	10	Previous lung adenocarcinoma; CIHD; DM II; endocrine/metabolic disorders; urinary *E. coli* infection
12	60	6	5	2	3	4	Valvular disease; dyslipidemia
13	60	17	4	4	11	12	AF, AH; DM II; COPD/OSAS; previous colon cancer
1 4	45	7	5	2	3	4	Extrasystoles, valvular disease; osteoarthritis; hypothyroidism
15	50	17	9	3	6	7	Bradycardia, hypertensive/valvular disease; COPD; DM II; hepatic steatosis
16	50	16	12	2	9	10	DM II, hypothyroidism; AV block/pacemaker, valvular disease; COPD; CKD IIIB; PAD; previous bladder cancer; CRE rectal colonization
17	65	9	6	1	0	1	AH; mild valvular disease
18	65	10	5	1	6	8	Previous breast cancer
19	55	9	9	3	8	10	AH; RA; previous TIA; COPD; previous bladder cancer
20	45	8	5	2	0	2	AF, extrasystoles, mild valvular disease; atherosclerotic vascular disease; osteoarthritis; CRE rectal colonization
21	65	11	9	2	6	9	CKD IIIA; AH/aortic stenosis; DM II; liver fibrosis; CRE rectal colonization

All abbreviations, including those referring to comorbid conditions, are defined in Table 3.

**Table 5:** Treatment characteristics, clinical responses, and toxicity profile.

Parameter	Value	Parameter	Value
Pts treated with AZA+VEN (*N°*)	21/21	Median cycles, *N°* (range)	7 (1–29)
Median cycles to CR (*N°*)	2	Median time to response (months)	1.8
Relapsed patients	5/21 (23.8%)	Median time to relapse (months)	15.6
Alive at last FUP (*N°*)	10/21 (47.6%)	Ongoing treatment (*N°*)	7
Dead (*N°*)	11/21 (52.4%)	AML-related deaths (*N°*)	5/11 (45%)
		Non-AML-related deaths	6/11 (55%)
Wash-out performed (*N°*)	5/21 (23.8%)	Median days of wash out	7
G-CSF administration	17/21 (81.0%)	Median G-CSF duration (days)	5.5
**Response**	***N°*** **= 12/21 (57.1%)**		
**CR/CRi**	**9/21 (42.9%)**	**MLFS**	**2/21 (9.5%)**
▪ CR	3/21 (14.3%)	**PR**	**1/21 (4.8%)**
▪ CRi ▪ MRD-neg	6/21 (28.6%) 2/9 (22.2%)		
**Non responders**	*N°* = 4/21 (19.0%)	**Stable disease**	5/21 (23.8%)
**AZA schedule modification**	***N°*** **= 12/21 (57.1%)**	**Reason**	***N°***
5-day schedule from cycle ≥ 2	12/21 (57.1%)	Remission achieved Other reasons	8 4
No modification	9/21 (42.9%)	/	/
**VEN schedule modification**	***N°*** **= 14/21 (66.7%)**	**Reason**	***N°***
14 days, 200 mg from cycle ≥ 2	7	Modification due to remission	11
14 days, 300 mg from cycle ≥ 2	3	Modification due to other reasons	3
14 days, 400 mg from cycle ≥ 2	2	No modification	7
15 days, 400 mg from cycle 1	1		
100 mg from 1st cycle (posaconazole)	1		
**Hematologic toxicities/patient**	***N°***	**N° of non-hematologic toxicities/patient**	***N°*** **/%**
Anemia Thrombocytopenia Neutropenia	21/21(100%)	1 toxicity 2 toxicities 3 toxicities	1 (11/52%) 2 (10/48%) 3 (5/24%)

Abbreviations: AZA (azacitidine); VEN (venetoclax); AML (acute myeloid leukemia); FUP (follow-up); G-CSF (granulocyte colony-stimulating factor); CR (complete remission); Cri (complete remission with incomplete hematologic recovery); MRD (measurable residual disease); MLFS (morphologic leukemia-free state); PR (partial remission). Bold text identifies the main variable categories in the table.

**Table 6:** Treatment exposure, dose and schedule modifications, treatment interruptions, and supportive management during frontline AZA+VEN therapy.

Treatment Characteristic	*N°* (%) Value	Timing	Main Reason/ Clinical Context
**Initial Treatment**			
AZA 75 mg/m^2^, 7-day/28-day cycle	21/21 (100%)	Cycle 1	Standard starting schedule
VEN ramp up: 100 mg/die + 1200 mg/die + 2400 mg/die + 3	21/21 (100%)	Cycle 1	Standard starting schedule
CYP3A-interacting azole (posaconazole) requiring VEN adjustment (100 mg/die)	1/21 (4.8%)	Cycle 1–2	Patient with chronic respiratory disease and higher risk of lung mitotic infections
**Azacitidine Modifications**			
Any AZA schedule modification	12/21 (57.1%)	Cycle ≥ 2°	Response-adapted treatment or clinical/tolerability considerations
AZA shortened from 7 to 5 days	12/21 (57.1%)	Cycle ≥ 2°	Mainly after achievement of response
Modification after response/remission	8/12 (66.6%)	Median, 2 cycles	Avoid cumulative treatment burden Avoid myelosuppression
Modification for reasons other than response	4/12 (33.3%)	1 from 4° cycle 1 from 3° cycle 1 from 5° cycle 1 on 5–9° cycles	Reduced tolerability (nausea, infections)
No AZA schedule modification	9/21 (42.9%)	—	Generally good tolerability Patients not in remission
**Venetoclax Modifications**			
Any VEN schedule/dose modification	14/21 (66.7%)	Predominantly from cycle ≥ 2	Mainly response-adapted; hematologic recovery; comorbidity; concomitant therapy; tolerability
VEN 200 mg for 14 days	7/21 (33.3%)	From cycle ≥ 2	Individualized post-response schedule In order to avoid prolonged cytopenia
VEN 300 mg for 14 days	3/21 (14.3%)	From cycle ≥ 2	Individualized post-response schedule Reduced gastrointestinal tolerance
VEN 400 mg for 14 days	3/21 (14.3%)	From cycle ≥ 2	2 pts underwent an adapted treatment schedule in the setting of post-MDS AML; 1 pt requested to reduce oral medication burden post-remission due to concomitant polypharmacy
VEN 100 mg with posaconazole	1/21 (4.8%)	From cycle 1	CYP3A-mediated drug interaction
No VEN modification	7/21 (33.3%)	—	Generally good tolerability Patients not in remission
**Treatment Delays/Interruptions** **Supportive Management**			
Temporary treatment interruption (“washout”)	5/21 (23.8%)	During subsequent cycles	Predominantly to permit hematologic recovery
Median washout duration	7 days	—	G-CSF administered in order to avoid further delay
Additional cycle delays documented in individual patients	1/21 (4.8%)	2°cycle	Hospitalization due to renal insufficiency
G-CSF administration	17/21 (81.0%)	During treatment	Neutropenia/accelerate hematologic recovery
Median G-CSF duration	5.5 days	—	—
**Treatment Exposure**			
Median number of AZA+VEN cycles	7		
Received ≥ 1 cycle	21/21 (100%)	Received ≥ 2 cycles	17/21 (81.0%)
Received ≥ 3 cycles	12/21 (57.1%)	Received ≥ 4 cycles	10/21 (47.6%)
Received ≥ 5 cycles	9/21 (42.9%)	Received ≥ 6 cycles	7/21 (33.3%)
Received ≥ 10 cycles	4/21 (19.0%)	Received ≥ 14 cycles	3/21 (14.3%)
Received ≥ 20 cycles	2/21 (9.5%)	—	—
**Permanent Discontinuation**	9/21 (42.9%)	Mainly from cycle 2	Refractory/progressive AML, deterioration of general condition, persistent cytopenia, intercurrent comorbidity/infection, death

Abbreviations: Pt/Pts (patient/patients); CYP3A (cytochrome P450 3A); G-CSF (granulocyte colony-stimulating factor); MDS (myelodysplastic syndrome). Bold text identifies the main variable categories in the table.

**Table 7:** Hematologic and non-hematologic toxicities observed during AZA+VEN treatment.

**Hematologic Toxicity**	**Grade 1**	**Grade 2**	**Grade 3**	**Grade 4**
Anemia	2	6	12	1
Thrombocytopenia	1	1	4	12
Neutropenia	1	1	4	15
**Non-Hematologic Toxicity (*N°tot* = 28)**				
**Infection-related (*N°* = 14/28—50%)**	**Grade 1**	**Grade 2**	**Grade 3**	**Grade 4**
Febrile neutropenia	0	1	1	0
Respiratory tract infections	0	3	1	0
Urinary tract infections	0	1	3	0
Herpes zoster infection	0	1	0	0
Influenza A	0	1	0	0
PICC infection	0	1	0	0
Diarrhea (*C. difficile*)	0	0	1	0
**G.l. Symptoms (*N°* = 5/28—17.9%)**				
Nausea/Vomiting	0	2	0	0
Diarrhea	3	0	0	0
**Laboratory abnormalities** **(*N°* = 4/28—14.3%)**				
Electrolyte disturbances (hypokale-mia/hyponatremia/hypocalcemia)	2	0	1	0
Increased amylase/lipase	1	0	0	0
**Cardio-respiratory (*N°* = 3/28—10.7%)**				
Atrial fibrillation/flutter	0	0	2	0
Bronchial stricture	0	1	0	0
**Others (*N°* = 2/28—7.1%)**				
Fatigue	0	0	1	0
Skin allergic reaction	0	1	0	0
**Safety Outcome (overall cohort *N°* = 21)**		***N°*** **/%**	**Details**	
Grade 4 infections		0/21 (0%)	—	
Febrile neutropenia		2/21 (9.5%)	ANC < 100/mm^3^	−T 38–39 °C
Tumor lysis syndrome		0/21 (0%)	—	
Significant bleeding		1/21 (4.8%)	Other	causes
Unplanned hospitalization		2/21 (9.5%)	Other	causes
Treatment-related mortality		0/21 (0%)	—	

Abbreviations: PICC (peripherally inserted central catheter); GI (gastrointestinal); *C. difficile* (*Clostridioides difficile*); ANC (absolute neutrophil count); T (temperature). Bold text identifies the main variable categories in the table.

**Table 8:** Patient-level characteristics, treatment exposure, and outcomes of patients with AML secondary to MDS who have been previously exposed to AZA before AZA+VEN.

Patient	Prior AZA Cycles	Molecular and Cytogenetic Features	Best Response to AZA+VEN	Post-Response AZA+VEN Schedule	AZA+VEN Treatment Duration	Clinical Outcome
1	1	*TP53* VAF 43% Complex/monosomal karyotype	MLFS after 1 cycle	- AZA 5 days; - VEN 300 mg 14 days	6 cycles	TI from cycles 2 to 4. Discontinued after cycle 6 due to cytopenia and AML relapse. Alive, receiving supportive therapy
2	1	*TP53* VAF 93% Complex karyotype	Not evaluable	NA	2 cycles	Discontinued because of clinical deterioration; died due to progressive AML
3	8	NPM1 mutated Karyotype 46, XY	Refractory	NA	2 cycles	Died due to progressive AML
4	5	*ZRSR2* VAF 92%; *RUNX1* VAF 29%; *EZH2* VAF 44%; Karyotype 46, XY	CR after 2 cycles	- AZA 5 days - VEN 200 mg 14 days	4 cycles (ongoing)	Alive CR TI from cycle 2
5	7	No pathogenic mutation detected Karyotype 46, XY	CR after 2 cycles	- AZA 5 days - VEN 200 mg 14 days	14 cycles	Alive after disease relapse; currently receiving HU

Abbreviations: AML (acute myeloid leukemia); MDS (myelodysplastic syndrome); AZA (azacitidine); VEN (venetoclax); CR (complete remission); MLFS (morphologic leukemia-free state); VAF (variant allele frequency); TI (transfusion independence); NA (not applicable); HU (Hydroxyurea).

## Data Availability

The data supporting these findings are available on reasonable request to the corresponding author. Data are not publicly available due to privacy or ethical restrictions.

## Abbreviations

The following abbreviations are used in this manuscript:

ADL	Activities of Daily Living
AF	Atrial Fibrillation
Allo-SCT	Allogeneic Stem Cell Transplantation
AML	Acute Myeloid Leukemia
AZA	Azacitidine
BCL-2	B-cell Lymphoma 2
CBC	Complete Blood Count
CCI	Charlson Comorbidity Index
CHR	Complete Hematologic Recovery
CHT	Chemotherapy
CIHD	Chronic Ischemic Heart Disease
CIRS	Cumulative Illness Rating Scale
CMML	Chronic Myelomonocytic Leukemia
COPD	Chronic Obstructive Pulmonary Disease
CR	Complete Remission
CRi	CR with Incomplete Hematologic Recovery
CRE	Carbapenem-Resistant Enterobacterales
CTCAE	Common Terminology Criteria for Adverse Events
CKD	Chronic Kidney Disease
DM	Diabetes Mellitus
ECOG-PS	Eastern Cooperative Oncology Group Performance Status
ELN	European LeukemiaNet
FU	Follow-Up
G-CSF	Granulocyte Colony-Stimulating Factor
GI	Gastrointestinal
HCT-CI	Hematopoietic Cell Transplantation–Comorbidity Index
HHD	Hypertensive Heart Disease
HMA	Hypomethylating Agent
HR	Hematologic Response
ITP	Immune Thrombocytopenia
M	Male
MDS	Myelodysplastic Syndrome
MFI	Myelofibrosis
MLFS	Morphologic Leukemia-Free State
MPN	Myeloproliferative Neoplasm
MR	Myelodysplasia-Related
MRD	Measurable Residual Disease
NGS	Next-Generation Sequencing
NR	Non-Responder/Not Reached
ORR	Overall Response Rate
OS	Overall Survival
PAD	Peripheral Artery Disease
PFS	Progression-Free Survival
PLT	Platelet Count
PR	Partial Remission
PS	Performance Status
RT	Radiotherapy
RT-PCR	Real-Time Polymerase Chain Reaction
SD	Stable Disease
SOC	Standard of Care
TB	Tuberculosis
TIA	Transient Ischemic Attack
VAF	Variant Allele Frequency
VEN	Venetoclax
WHO	World Health Organization
